# Prevalence of Plantar Warts, Genital Warts, and Herpetic Infections in Greek Competitive Swimmers

**DOI:** 10.3390/v16111782

**Published:** 2024-11-16

**Authors:** Eleni Sfyri, Niki Tertipi, Vasiliki Kefala, Efstathios Rallis

**Affiliations:** Department of Biomedical Sciences, University of West Attica, Campus I, 12243 Athens, Greece; ntertipi@uniwa.gr (N.T.); valiakef@uniwa.gr (V.K.); erallis@uniwa.gr (E.R.)

**Keywords:** viral skin infections, competitive swimmers, plantar warts, herpetic infections, sexually transmitted viruses

## Abstract

Viral outbreaks are common in the sport community. Data regarding the prevalence of plantar warts, genital warts, herpes simplex type 1 (herpes labialis), herpes zoster, and genital herpes in competitive swimmers are lacking in the literature. The purpose of this study was to determine the prevalence of those viral infections among young competitive swimmers participating in Greek swimming clubs. Swimmers’ parents and adult swimmers were asked to complete an anonymous questionnaire. In total, 1047 swimmers enrolled in this study. The measured parameters included gender, age, times of infections, and seasons when athletes may be more susceptible to infections. Practicing information such as type of swimming facility, number of training years, average hours of daily training, behaviors in swimming practice, and sunlight exposure was also recorded. All infections showed a significant difference in relation to “age” and “years of training”. The gender significance was observed in herpes labialis (*p* = 0.016) and plantar warts (*p* = 0.05). The prevalence of all infections in swimmers who use outdoor facilities was higher. Certain behaviors such as walking barefoot on a pool deck and sharing swimming equipment correlate with herpes simplex and plantar warts. Virus infections can affect swimmers of all ages. In our study, plantar warts and herpes labialis are more common in swimmers. Herpes zoster and sexually transmitted viruses are rarer and affect adult swimmers. The impact of cutaneous infections on swimmers can affect performance and well-being. Effective prevention and management are essential to avoid complications. Proper hygiene, medical guidance, and treatment reduce swimmers’ exposure to skin viruses.

## 1. Introduction

Cutaneous infections are common in both the general population and among athletes [1]. Swimming, especially popular among children and young adults, carries a notable risk of skin infections [2]. Exposure to moisture, heat, cold, wind, chemicals, and sunlight (especially in open pools) contributes significantly to the incidence of skin conditions in swimmers [3,4]. Certain viral cutaneous infections are particularly prevalent, with primary risk factors including the pool environment, equipment, and swimmers’ routines [5,6]. Moreover, skin viruses that are generally prevalent in the wider population—often driven by external factors—could disrupt training programs for swimmers and lead to more serious health concerns.

Warts are benign proliferative lesions of the skin and mucous membranes caused by the human papillomavirus (HPV). There are 231 types of HPV [7]. Common warts are primarily associated with HPV types 2 and 4. Plantar warts are commonly caused by infection with types 1, 2, 4, 60, or 63. Deep palmoplantar warts are caused by type 1, cystic warts by type 60, and focal epithelial hyperplasia by types 13 and 32 [4]. Type 6 is responsible for genital warts. Warts are widespread across the general population, affecting both children and adults, with a prevalence ranging from 4% to 33% [2,3]. The disease is globally prevalent and is transmitted through direct contact with infected skin or indirectly via contaminated objects [2,8]. A common form of warts seen in swimmers is plantar warts. Swimmers are at a significantly higher risk of developing these compared to the general population [6]. Certain habits, such as not wearing footwear on poolside surfaces and the use of communal showers, contribute significantly to the risk of infection [6,9,10]. Genital warts are also highly prevalent in the global population, representing the most common sexually transmitted infection worldwide. The prevalence rate of HPV is challenging to determine due to the frequently asymptomatic nature of the infection; however, the risk of being infected at least once in a lifetime among both men and women is 50% [11]. In the last two decades, vaccines have been developed and approved for HPV, aiming to prevent infections it causes and the diseases associated with them, such as cervical cancer, anal cancer, other types of cancer, and genital warts.

Other common viral skin infections are those caused by the herpes simplex virus (HSV). This virus has a global distribution, affecting all populations and impacting both individual and public health. Once infection occurs, it remains lifelong with intermittent clinical and subclinical reactivation [12]. The herpes simplex virus has two main types. Type 1 (HSV-1) is primarily transmitted through oral contact and causes infections in and around the mouth (herpes labialis), but it can also cause genital herpes through oral–genital contact. Herpes labialis infection affects both sexes and all age groups, with humans being the sole host of the virus [13]. Type 2 (HSV-2) is the leading cause of genital herpes and is a sexually transmitted infection. The prevalence of HSV increases with the onset of sexual activity during adolescence and continues to rise steadily into adulthood. Women are more susceptible to this infection than men. Over the past two decades, HSV has been associated with a threefold higher risk of HIV (Human Immunodeficiency Virus) transmission [13,14]. Most individuals infected with either type experience no symptoms or only mild symptoms. Prolonged exposure to ultraviolet radiation, especially during the summer months, increases the risk of HSV reactivation in both the general population and athletes. During the summer, approximately 20% of the population may experience HSV infection, with 40% of these cases occurring in individuals under the age of 30 [13].

Herpes zoster is a viral and painful skin infection caused by the reactivation of the varicella-zoster virus (VZV). It is initiated by chickenpox, acquired typically in early childhood. The disease affects both sexes, with an incidence in the general population of approximately 10–20%, increasing to 50% in individuals aged 85 and older. It manifests in about 10% of those under 20 years of age and 5% of individuals younger than 15 [15]. It is estimated that roughly 1 in 4 people will develop herpes zoster during their lifetime. Vaccination is recommended for older populations. Varicella vaccines have become part of routine immunization programs in many countries, making childhood vaccination a potential factor in preventing herpes zoster later in life and decreasing the incidence in the long term [16,17]. As with other herpes infections, outbreaks may be triggered by prolonged sun exposure [18].

Research on dermatological conditions specific to swimming has been limited, often consisting of isolated reports or reviews rather than comprehensive studies with large swimmer cohorts [3,4]. The manifestations of common viral infections in swimmers, such as plantar warts and herpes labialis, are primarily limited to older reports and lack recent data. Furthermore, there are no literature references regarding genital warts, genital herpes, or herpes zoster in swimmers. Investigating the presence of these viral infections in competitive swimmers is crucial for their prevention and protection against the spread of these pathogens, as well as for mitigating the potential impacts on training schedules and competition processes.

## 2. Materials and Methods

This cross-sectional study was designed to investigate the prevalence of skin infections among competitive swimmers in Greece, considering the influence of the COVID-19 pandemic on data collection. The researchers obtained necessary ethical approvals from the University of West Attica (52645-20 July 2020) and the Hellenic Swimming Federation (787-15 March 2019). Due to restrictions during the pandemic, an online survey was conducted between June and December 2021. The methodology used was based on self-selected sampling, which was the only method that could be applied due to the difficulty of reaching the entire competitive population of Greek swimmers.

The study involved participants from swimming clubs all over Greece. From a total of 182 swimming clubs, swimmers from 80 clubs responded. The recruitment process for participants involved sending invitations to adult swimmers and the parents of underage swimmers, through email and social media, with coaches and managers of the respective clubs facilitating communication. Participation was voluntary and anonymous. A total of 1047 individuals participated, all of whom were competitive swimmers, from junior categories (9–12 years old), age group categories (13–18 years old), and men–women category. The collected sample (9.23%) was considered statistically satisfactory and representative of the total population of swimmers.

The questionnaire was designed and validated by the researchers through a pilot study where the test–retest method was provided. It was administered via Google Forms and divided into two main sections. The first section covered general information, including demographics, training routines, behavior in the pool area, and overall skin health. The second section focused on specific details regarding various infections. For this study, data from specific sections of the questionnaires were used, focusing on demographic characteristics, training routines, times of infections, and the season when athletes may be more susceptible to infections. Practicing information such as the type of swimming facility, number of training years, hours of daily training, and behaviors in swimming practice was also recorded. For recurrent infections, participants were asked to provide information about the most recent episode, as it is typically easier for respondents to recall more recent events accurately.

### Statistical Analysis

The categorical variables were presented as absolute (*n*) and related frequencies (%). The chi-square test was used to examine the relationship between two categorical variables. For investigating relationships between a categorical variable and an ordinal variable, the chi-square trend test was used. The bivariate correlations were used between virus infections and “gender”, “type of facility”, “years of training”, and “hours of daily training”. Correlations were made between the two different types of warts and behaviors of swimmers, such as “walking barefoot around the pool area” or “share equipment”. In the case of herpes infections, correlations were drawn with factors like “sun exposure”, “use of sunscreen”, and “sun burn”.

Multivariate logistic regression analyses were conducted to identify potential associations between, hygiene habits (e.g., sharing equipment and behaviors), time of the daily training, sunscreen use and sunburn, and the viruses’ s infections under study. This was achieved by calculating the odds ratio (OR) along with a 95% confidence interval (CI). The level of statistical significance (*p*) was set at 0.05 for all tests (two-sided). Data analysis was performed using IBM SPSS 26.0 software (Statistical Package for Social Sciences).

## 3. Results

### 3.1. Demographic Characteristics

One thousand and forty-seven swimmers (*n* = 1047) were enrolled and participated in this study. The response rate was 9.23%. Overall, 577 were females (55.1%) and there were 470 males (44.9%) among the respondents. In total, 637 swimmers used an outdoor facility (60.8%) for their training and 470 swimmers (39.2%) used an indoor facility. The majority of participants belonged to the junior categories (9–12 years old) (*n*=359, 34.3%) followed by the age group categories “13–14 years old” (*n*=231, 22%), “15–16 years old” (*n* = 195, 18.6%), “17–18 years old” (*n* = 111, 10.6%), and “>18 years old” (*n* = 151, 14.4%). The training years for most participants were “7–9 years” (*n* = 265, 25.3%) and “4–6 years” (*n* = 262, 25%). According to parents’ and swimmers’ responses, half of the swimmers (*n* = 541) have a two-hour daily training schedule.

### 3.2. Wart Infections

#### 3.2.1. Plantar Warts

A total of 382 participants (36.5%) experienced plantar warts at some point during their swimming careers. Nearly half of the plantar wart infections occurred during the winter, showing a significant difference compared to those infected in other seasons (*n* = 158, 41.4%, *p* < 0.001). Infections were significantly less common in the spring (20.1%, *p* < 0.001), summer (14.1%, *p* < 0.001), and autumn (11%, *p* < 0.001). The training was interrupted for 212 swimmers (55.5%) during their plantar wart treatment, though the duration of absence varied. Meanwhile, 170 swimmers continued their training while receiving treatment. Almost one-third of the infected swimmers (*n =* 103 26.9%) had an absence of “less than a month”, 32 swimmers (8.2%) were absent for “less than three months”, and 70 swimmers (18.3%) were absent for “less than a week”. In three cases, swimmers had to stop training for more than six months due to plantar warts. Four swimmers did not answer this question (Table 1).

In all instances, the infection was diagnosed and treated by dermatologists. Although more females were affected, the prevalence between genders showed no significant difference (*p* = 0.085). While younger swimmers made up a larger portion of the group, older participants were more likely to contract plantar warts. Specifically, 43.7% of adults and 40.5% of swimmers aged 17–18 had warts, compared to 31.5% of those aged 9–12 and 35.1% of those aged 13–14. Most of the infected swimmers used an open facility. The number of years spent swimming and the length of daily training sessions appear to influence wart infection rates. In particular, training for more than two hours daily was the most common among swimmers, which likely contributed to the higher incidence of plantar warts (Table 2).

Swimmers who walk barefoot on the pool’s deck had an increased risk of having plantar warts (*n* = 297, 77.7%) (OR = 1.535 CI 95% 1.146–2.056, *p* = 0.004). Wearing flip-flops was a hygiene practice followed by nearly all participants. However, placing bathrobes or towels on the pool’s bench (*n* = 272, 71.2%) (OR = 1.471, 95% CI: 1.121–1.929, *p* = 0.005) and sharing fins (*n* = 172, 45%) (OR = 1.559, 95% CI: 1.206–2.017, *p* = 0.001) were behaviors that significantly increased the risk of developing plantar warts.

#### 3.2.2. Genital Warts

Genital warts were reported by 1.3% (*n* = 14). In the adult population, the rate was 9.3%. Symptoms lasted “less than 3 months” for nine swimmers. The disease was reported to manifest during the summer by eight swimmers. None of the swimmers interrupted their training sessions (Table 1). Genital warts were reported by six (7.6%) men and eight (11.1%) women. Eleven of the swimmers used an open facility and all of them had been training in swimming for more than 10 years (Table 2).

### 3.3. Herpetic Infections

#### 3.3.1. Herpes Labialis

Overall, 8.2% of the participants reported having contracted herpes labialis. Among them, 51.2% had it “once” and 17.4% “more than six times”. The reported seasons of infection were “winter” (40.5%) and “spring” (23.8%). Of those affected, 50% “did not interrupt their training”, while 47.4% stopped for “less than a week”. (Table 3). Additionally, 52.6% did not visit a dermatologist, while 21.1% reported that they self-administered medication.

The correlations between demographic characteristics and training variables with the occurrence of herpes labialis showed that female swimmers (10.1%) had a higher rate of infection, with a statistically significant difference (*p* = 0.016). The majority of those infected were using an open facility. Adult swimmers and those aged “15–16 years” also exhibited a higher percentage of herpes labialis. Similarly, years of swimming experience (*p* < 0.001) and more hours of training appear to be associated with the occurrence of herpes labialis (Table 4). Swimmers who use swimming puddles (*n* = 51,12.4%) had an increased risk of having herpes labialis (OR 2.346 CI 95% 1493–3687 *p* < 0.001).

The study examined the relationship between the occurrence of herpes infections, training time, sunscreen use, and sunburn. Herpes labialis appears to be associated with training during hours of sun exposure (*p* = 0.007), non-use of sunscreen (*p* = 0.008), and sunburn (*p* < 0.001). Additionally, the data show that the number of swimmers affected by herpes labialis who train in an open facility coincides with those who train between ‘12:00–17:00′ and have double training sessions (Table 5). Further statistical investigation, considering the significance observed in the aforementioned associations, revealed that non-use of sunscreen (OR 1.493 CI 95% 1.011–2.205 *p* = 0.044) and sunburn (OR 3.156 CI 95% 1.759–5.661 *p* < 0.001) are associated with an increased risk of developing herpes labialis.

#### 3.3.2. Genital Herpes

Genital herpes was reported by 1.2% (*n* = 13) of the total participants. All responders were adults and in the adult population analysis, the rates were 8.6%. The season of herpes manifestation was mainly “summer”, followed by “autumn” and “winter”. Almost half of the participants who had been infected did not interrupt their training. Five did not answer this question (Table 3). Genital herpes was reported by eight male swimmers and five female swimmers. Nine of the participants used an open facility, and all of them had been training in swimming for more than 10 years (Table 4). Occasional use of sunscreen and a history of sunburn were some of the characteristics observed in swimmers who developed genital herpes (Table 5). Further statistical investigation revealed that the likelihood of developing genital herpes is 14 times greater for those who have experienced sunburn (OR 14.103 CI 95% 4.628–42.979 *p* < 0.001).

#### 3.3.3. Herpes Zoster

Overall, 1.9% (*n* = 20) of the participants contracted herpes zoster. Half of the participants who developed herpes zoster did not report a specific season of onset. The most commonly reported seasons of onset were “winter” (15%) and “autumn” (15%) (Table 3). The majority of participants who exhibited herpes zoster (7.3%) were adult swimmers, with a small percentage reported among younger age groups (*p* = 0.005). Reports of herpes zoster were limited, with women exhibiting it more frequently (*n* = 12). The majority of those infected were using an open facility (*n* = 16), trained during sunny hours, during the period “12:00–17:00”, participated in double training sessions (Table 4), and had also experienced sunburn in the past (11.6%) (Table 5). Further statistical investigation revealed that the likelihood of developing herpes zoster is 12 times greater for those who have experienced sunburn (OR 12.513, CI 95% 5.051–30.997, *p* < 0.001). All participants who contracted the infection followed a treatment regimen prescribed by a dermatologist.

## 4. Discussion

Viral skin infections have become more prevalent as the number of athletes has risen. [1]. For this reason, in this study, we aimed to address viral infections that may occur in competitive swimmers, some of which are attributed to the swimming pool environment and the habits of swimmers, while others mainly are caused by external factors. Based on our findings, the incidence of plantar warts among Greek swimmers is considered high [19], while the incidence of herpes labialis virus is lower than in the general population, but double compared to the results of another study [20,21].

Herpes zoster, genital herpes, and genital warts were investigated as viral infections that, according to the literature, are not associated with participation in swimming. The rates observed in our study were lower than those reported in the general population [22,23,24]. The literature is lacking concerning research on swimmers, making it difficult to compare infection rates between studies. In other studies, the most commonly reported sports-related infections were fungal, bacterial, contact dermatitis, and common viral infections [1,25,26]. To our knowledge, this is the first study to present the epidemiology of skin infections among a large number of competitive swimmers. The previous literature on swimmers’ skin infections has been limited to a small number of participants and literature reviews [3,4,5,6,9,27].

The global prevalence of warts, regardless of location, is reported to be at 6% in children and 3–20% in the general population. Plantar warts occur most frequently in children and adolescents, although they are rare in patients younger than 5 years old [19]. In a preliminary study conducted on Greek swimmers, 43% of participants reported having warts, regardless of location [6]. In the present study, the occurrence of plantar warts among our athletes was significantly higher (36.5%) compared to the 7–12% found in the general population [19,28]. The soles are common sites of infection for swimmers due to the use of communal showers, walking barefoot around pool areas, microtrauma to the skin from these behaviors, and failure to follow hygiene protocols [29]. In our study, despite the widespread use of slippers, a strong correlation was found between the occurrence of plantar warts and certain habits among swimmers, including walking barefoot around the pool, placing towels on benches, and sharing equipment such as flippers. These findings align with those reported in other studies [6,10].

Female swimmers appear to develop plantar warts more frequently than male swimmers, a result that differs from the findings of other studies, where a higher frequency was reported in male children [30,31]. The high prevalence rate from the age of 13 and above, as seen in our results, is related to the years and duration of training [6,31]. It is noteworthy that 44.5% of swimmers with plantar warts did not interrupt their training, as they should have. This practice was linked to covering the infected feet with special socks, a recommendation also mentioned in the Health Department’s guide for schools in the United Kingdom, allowing affected individuals to participate in swimming lessons. However, the validity of this practice has not been scientifically confirmed [32]. The rise in plantar warts during the winter and spring among our swimmers is corroborated by another study, which attributes this increase to the intensive training that occurs during these seasons [28]. The competitive swimmers’ training period is from autumn to early summer and the prevalence of warts in winter and springtime are justified considering the daily attendance in the swimming pool. On the other hand, Tamer et al. found that warts were detected more in summer than wintertime [33].

An outdoor facility may have a higher risk of virus transmission due to exposure to the open-air environment. However, Vaile et al. reported a greater prevalence of plantar warts in swimmers in indoor facilities compared to outdoor, concluding that this difference was observed during different periods of exposure [32]. The role of bathing areas, such as indoor swimming pools or amusement parks, in the spread of other cutaneous infections has been well established [34]. Regarding the occurrence of skin infections, further research is needed to assess the influence of environmental factors.

Sexual activity is a widely discussed topic across various medical disciplines due to its significant impact on health status [35]. It is well-established that up to 70% of both sexes in the global population will be infected with sexually transmitted infections (STIs) at some point in their lives [36]. Two sexually transmitted diseases were examined in this study to determine their prevalence among swimmers, an area of research that has not been previously explored.

The spectrum of genital HPV infections is extensive, encompassing a range of manifestations from genital warts to various forms of cancer [37]. This virus remains the main cause of cancer-related mortality among women [38]. Infections in men are often asymptomatic, but studies on the presence of HPV in males are increasing. The epidemiological distribution of HPV infection varies across different regions globally [39]. Socio-economic, geographical, and genetic factors are associated with genomic variability and morbidity, as well as individual factors such as gender, age, and overall health status. The prevalence of swimmers with genital warts was lower (9.3%) compared to the literature, where the rate reaches 13.8%, and there is a 50–80% global probability of exposure to and infection by the virus [22]. In the 2013–2014 period, the prevalence of anogenital HPV infection was estimated to be 42.5% among U.S. adults aged 18–59 years [40]. Over the past two decades in Europe, the prevalence of HPV has been steadily rising, with the United Kingdom experiencing an approximate 25% increase between 1996 and 2005 [41]. The peak incidence of HPV infections occurs between 20 and 29 years of age [41,42]. This observation is consistent with our study’s findings, where all the infected participants were between 18 to 26 years old.

Although in Greece, the vaccination for human papillomavirus (HPV) has been covered by the National Health System for the adolescent female population over the past two decades, and more recently for males, the number of female swimmers (*n* = 8, 11.1%) was slightly higher compared to male swimmers (*n* = 6, 7.6%). This may be due to the fact that the symptoms are asymptomatic, and most women visit a gynecologist more frequently for their annual check-ups. Conversely, previous studies have demonstrated that men are approximately five times more likely to be diagnosed with genital warts [36]. The risk of contracting the disease in young athletes is significant due to high-risk behaviors related to their sexual activity and the lack of awareness about the virus [43]. The literature showed that premature initiation of sex life and unprotected sex among young athletes contribute to the growth of genital warts [44]. For this sexually transmitted virus, there is a lack of specific data regarding swimmers, and the existing literature on athletes is limited, primarily addressing various forms of warts [45]. It is evident that further research is necessary to better understand the virology and pathophysiology of this infection, as well as clinical manifestations. Additionally, comprehensive public health campaigns should emphasize awareness of the available vaccines, which should be integrated into standard public health initiatives [43].

The prevalence of swimmers in our study who contracted herpes simplex virus was 8.2%, which is lower than the general population (15–20%) [21,46] but approximately double the 4.76% reported in swimmers [20,47]. Lower percentages were also observed in the swimmers of our study, aged 9–12 years (3.7%) and 13–18 years (ranging from 7.3% to 9.2%), while the rates in the general child and adolescent population are 11.75% and 16.87%, respectively [13]. Female swimmers exhibited herpes labialis infections more frequently than male swimmers, a finding that aligns with the results of Shulman et al. [13], although there was no significant difference in further analysis.

The recurrence of herpes labialis is common and can be triggered by various factors, including exposure to sunlight [48]. This factor was recorded in 9.4% of our swimmers who trained in an open facility and 9.6% of those who trained between 12.00 and 17.00 p.m. In our study, winter and spring were noted as the most frequent seasons for the occurrence of HSV, a finding that contrasts with the study by Ichihashi et al. [49], who reported that sunburn and sun exposure caused HSV in approximately 20% of the general population during the summer months [21,50]. Considering that the manifestation of the virus is very common among winter sports athletes [51], we can justify the corresponding occurrences in our swimmers during winter and spring, the majority of whom are exposed to intense ultraviolet radiation daily, due to the mild climate in Greece and for more than 8 h a week. The use of waterproof sunscreen, at least on days with intense sunshine, may reduce the recurrence of the virus [21].

Herpes simplex virus type 2 (HSV-2), which is primarily transmitted through sexual contact, is responsible for genital infections. Conversely, herpes simplex virus type 1 (HSV-1), typically transmitted during childhood through nonsexual contact, is the leading cause of orolabial herpes; however, it can also result in genital infections. The positivity rates for HSV-2 tend to rise during adolescence and young adulthood, coinciding with the initiation of sexual activity, and generally plateau around the age of 30 years [24,52].

In the United States, between 2005 and 2010, the prevalence of HSV-1 among individuals aged 14 to 49 years was reported to be 53.9%, while the prevalence of HSV-2 was 15.7% [24,53]. Notably, there are no references in the existing literature regarding the incidence of genital herpes specifically in athletes [54]; the available data pertain largely to the general population [24,53]. In our study, we found that 8.6% of adult swimmers reported a history of genital herpes, which is lower than the reported incidence range of 10% to 60% in the general population [15,27]. This discrepancy may be attributed to the reluctance of some individuals to disclose a personal history of sexually transmitted diseases, even in an anonymous context. All swimmers who reported a diagnosis of genital herpes were adults, indicating a connection between the occurrence of the infection and sexual practices. Notably, male swimmers reported higher prevalence rates (10.1%) compared to female swimmers (6.9%), which contrasts with the existing literature that typically indicates a higher prevalence among women [14].

Populations engaging in high-risk behaviors exhibit elevated prevalence rates of genital herpes. Specifically, prevalence is notably higher in HIV-positive populations compared to the general population and in homosexual men compared to heterosexual men. Furthermore, genital herpes is frequently asymptomatic, often going unnoticed [53], which may account for the lower percentage of reported cases among female swimmers or the limited responses from our participants [55]. The epidemiological distribution of HSV infections varies significantly across different regions of the world. In Australia, the seroprevalence is approximately 13% [56], whereas in the United States, it is reported at 25% [57]. In Europe, a 2016 study indicated that among females aged 15–49 years, the infection rate for HSV-2 was 10.7%, while for males in the same age group, it was 5.3%. Additionally, the prevalence of HSV-1 infection was found to be 60.6% among females and 40.1% among males aged 0–49 years [58]. The overall prevalence of HSV infection ranges from 7.1% among individuals aged 15–19 years to 28.1% among those aged 40–44 years. Notably, the most significant increase in prevalence occurs between the ages of 20 and 24 years, which aligns with the findings of our study [52,59].

As with herpes labialis, there is a correlation between sunlight exposure, season of year, and recurrence of genital herpes [50,60]. Although participants in this study reported experiencing HSV symptoms only once, the findings align with the existing literature, linking sun exposure, lack of sunscreen use, and the manifestation of genital herpes. The increase in individuals exhibiting genital herpes following sun exposure in July and August was 19.7% in the general population and 40% in patients under 30 with recurrent HSV episodes [49]. Studies conducted among patients with recurrent genital herpes have shown prolonged sun exposure as well as stress in the period preceding the outbreak [61]. Persistent stress is considered a significant predictor of HSV recurrence, with individuals experiencing chronic psychological stress more likely to encounter HSV reactivations, including genital herpes, compared to those with lower stress levels. Stress weakens the immune system’s ability to maintain viral latency, making reactivation more probable [61]. These findings could also be associated with the stress of competitive events and physical fatigue from training, both of which may weaken the immune system. Effective stress management strategies, such as mindfulness, adequate sleep, and psychological support, may help reduce the frequency of HSV recurrences by enhancing immune function.

Another viral infection examined in this study was herpes zoster (HZ), which was reported by 1.9% of participants, a percentage significantly lower than 10–20% in the general population, particularly among individuals over 60 years of age [23]. To our knowledge, there are no studies specifically addressing the occurrence of HZ in swimmers. The disease has been noted in a single case involving triathletes [62]. The demanding and challenging training regimens of high-level athletes, along with the stress they experience, may negatively affect their immune system, making them more susceptible to the manifestation of HZ. Vaccination is generally recommended for the elderly and is not advised for athletes [63]. Female swimmers in our study showed a higher likelihood of contracting the infection, a finding corroborated by the literature [23].

The age of our athletes was associated with the disease, as cases primarily involved adult swimmers, consistent with the literature regarding the general population [64]. The absence of Herpes Zoster among younger swimmers may be related to mandatory vaccination, although there is also a viewpoint that widespread vaccination in healthy children could lead to a decrease in natural immunity to the varicella-zoster virus, which is responsible for boosting the immune system in adults, exposing them to reactivation of the clinically latent virus [64]. However, several studies have concluded that there is an increasing trend in the incidence of HZ even before the implementation of the vaccination program [23].

As mentioned above, herpes infections can be triggered by prolonged exposure to sunlight [48,50]. This factor was recorded for 16 of our swimmers who trained in an open facility with 13 of them training between 12:00 and 17:00 p.m. or participating in double training sessions. A recent study found that ambient UV radiation exposure was associated with a higher risk of herpes zoster in men, but not in women. Additionally, a history of severe sunburn was linked to a modestly increased risk of HZ in both men and women, possibly due to immunosuppression resulting from overexposure to the sun [65].

However, this study has certain limitations. Due to the restriction measures imposed during the COVID-19 pandemic, data collection was conducted through an online questionnaire, without clinical examination. All data were self-reported, and participants were required to recall past medical conditions, which introduces the potential for recall bias. We chose this method as it provided a faster and more convenient approach for participants, allowing them ample time to reflect on their responses without external pressure. The questionnaire encompassed a broad spectrum of skin infections, which were not specifically addressed in this study. As a result, we cannot conclusively determine whether participants’ responses were based on actual experiences with the viral infections under investigation. Adjusting for age in relation to exposure to dermatological infections could be another interesting parameter, but it was not included in this study. Some studies have examined the relationship between age and exposure to dermatological infections using a recent time frame (e.g., the past 12 months) rather than a specific age range [66,67]. Other studies using questionnaires do not mention the relationship between age and exposure to dermatological viruses at all [25,51]. An important parameter that was not included in the survey questionnaire, due to the broader scope of investigating skin diseases, was the vaccination for genital warts. This omission did not allow us to study the level of participation in vaccination in relation to the occurrence of the disease.

Based on the data obtained from this study, there is a correlation between the most common viral infections examined and the swimming environment, as well as the habits and practices adopted by swimmers. Notably, most infected swimmers continued to participate regularly in training sessions during their treatment, which may facilitate the transmission of the virus to their teammates. This investigation into viral infections unrelated to participation in swimming represents a unique approach in the existing literature. It highlights a rare viral manifestation in young individuals, such as herpes zoster, particularly in athletes, where published reports are notably limited. Regarding sexually transmitted infections, this study provides an initial exploration of this sensitive topic within a population segment that is characterized by frequent interactions with peers of the same age and a cultural emphasis on aesthetically pleasing, fit bodies, which are risk factors for potential virus transmission. Recommendations should be made to modify behaviors in the pool environment to mitigate the transmission of viruses, as well as guidance on the importance of consulting a dermatologist or other healthcare provider for the prevention and early management of potential infections [68]. A complete medical record, including dermatological conditions, should be maintained by the national federation for every competitive swimmer, which is not currently required or provided by the World Aquatics Federation.

## 5. Conclusions

Despite the fact that this study examined viral infections with different characteristics and manifestations, the findings are unique concerning competitive swimmers. A high prevalence of plantar warts was observed among competitive swimmers in Greece; however, the incidence of herpes labialis was lower than that reported in the general population, potentially due to the restrictions imposed on this specific group of swimmers and the older age of the participants. Furthermore, for viral infections such as herpes zoster, genital herpes, and genital warts, although the available reports are limited, they provide unique insights within the context of research on athletes. An early diagnosis of all skin viruses must be made for every swimmer through dermatological examination. In this way, possible transmission to other swimmers or individuals will be prevented. Further research is required to determine the frequency of occurrence as well as the factors that influence the manifestation of the aforementioned viral infections in sports.

## Figures and Tables

**Table 1:** Plantar warts and genital warts.

	Plantar Warts n (%)	Genital Warts n (%)
Yes	382 (36.5)	14 (9.3) (1.3) *
No	665 (83.5)	151 (90.7) (98.7) *
Season of infection		
Winter	158 (41.4)	1 (7.1)
Spring	77 (19.9)	2.14.3)
Summer	54 (14.1)	8 (57.1)
Autumn	42 (11)	3 (21.4)
Number of infectious events reported		
One	221 (57.9)	14 (100)
Two	66 (17.3)	0
Three	72 (18.8)	0
Four	10 (2.6)	0
Five	2 (0.5)	0
≥than six	10 (2.6)	0
Training interruption		
Non training interruption	170 (44.5)	9 (64.3)
<1 week	70 (18.3)	0
<1 month	103 (26.9)	0
<3 months	32 (8.4)	0
>6 months	3 (0.8)	0

* Of the total of the participants.

**Table 2:** Bivariate analyses using plantar warts and genital warts as dependent variables.

		Plantar Warts			Genital Warts	
Characteristics	Yes n (%)	No n (%)	*p* Value	Yes n (%)	No n (%)	*p* Value
Gender			0.085 ^a^			0.878 ^a^
Male	160 (34.1)	310 (65.9)		6 (7.6)	73 (92.4)	
Female	222 (38.4)	356 (61.6)		8 (11.1)	64 (88.9)	
Age ^b^			0.083 ^b^			0.001 ^b^
9–12 years old	113 (31.5)	246 (68.5)		0	359	
13–14 years old	81 (35.1)	150 (64.9)		0	231	
15–16 years old	77 (39.5)	118 (60.5)			194	
17–18 years old	45 (40.5)	66 (59.5)		0	112	
>18 years old	66 (43.7)	85 (56.3)		14	137	
Swimming pool			0.098 ^a^			0.533 ^a^
Outdoor facility	245 (38.5)	392 (61.5)		9(1.4)	628 (98.6)	
Indoor facility	137 (33.4)	273 (66.6)		4(1)	406 (99)	
Number of training years			0.001 ^b^			0.003 ^b^
≤3 years	27 (27)	73 (64.7)		0	100 (100)	
4–6 years	75 (28.6)	187 (71.5)		0	262 (100)	
7–9 years	115 (43.4)	150 (56.6)		2(0.8)	263 (99.2)	
10–12 years	83 (38.4)	133 (61.6)		4(1.9)	212 (98.1)	
>12 years	33 (36.3)	58 (63.7)		8(3.9)	196 (96.1)	
Hours of daily training			0.205 ^b^			0.896 ^b^
≤1.5 h/day	102 (32.9)	208 (67.1)		4(1.3)	306 (98.7)	
2 h/day	203 (37.5)	339 (62.5)		8(1.5)	535 (98.5)	
>2 h/day	77 (39.5)	118 (60.5)		2(1)	192 (99)	

Values are expressed as n (%), unless stated otherwise. ^a^ X^2^ test, ^b^ X^2^ test for trend.

**Table 3:** Herpetic infections.

	Herpes Labialis n (%)	Herpes Zoster n (%)	Genital Herpes n (%)
Yes	86 (8.2)	20 (1.9)	13 (1.2)/(8.6) *
No	961 (91.8)	1027 (98.1)	1034 (98.8)/151 (91.4) *
Season of infection			
Winter	34 (39.5)	3 (15)	3 (23.1)
Spring	20 (23.2)	1 (5)	1 (7.7)
Summer	14 (16.3)	2 (10)	4 (30.1)
Autumn	16 (18.6)	3 (15)	3 (23.1)
No answer	2 (2.3)	11 (55)	2 (15.3)
Number of infectious events reported			
One	44 (51.1)	20 (1.9)	13 (100)
Two	11 (12.8)	0	0
Three	10 (11.6)	0	0
Four	10 (11.6)	0	0
Five	2 (1.3)	0	0
≥Six	10 (11.6)	0	0
Training interruption			
No training interruption	43 (50)	2 (10)	6 (46.1)
<a week	41 (47.4)	0	2 (15.4)
<a month	2 (2.6)	2 (10)	0
No answer	-	16 (80)	5 (38.5)

* Of the adult participants.

**Table 4:** Bivariate analyses using herpes labialis, herpes zoster, and genital herpes as dependent variables.

	Herpes Labialis			Herpes Zoster			Genital Herpes		
Characteristic	Yes n (%)	No n (%)	*p* Value	Yes n (%)	No n (%)	*p* Value	Yes n (%)	No n (%)	*p* Value
Gender			0.016 ^a^			0.657 ^a^			0.225 ^a^
Male	28 (6)	442 (94)		8 (1.7)	462 (98.3)		8(1.7)	462 (98.3)	
Female	58 (10.1)	519 (89.9)		12 (2.1)	565 (97.9)		5(0.9)	572 (99.1)	
Age ^b^			<0.001 ^b^			<0.001 ^b^			<0.001 ^b^
9–12 years old	10 (3.3)	290 (96.7)		1 (0.3)	358 (99.7)		0	359 (100)	
13–14 years old	17 (7.3)	214 (92.7)		5 (2.2)	226 (97.8)		0	231 (100)	
15–16 years old	19 (9.8)	175 (90.2)		3 (1.6)	191 (98.4)		0	194 (100)	
17–18 years old	8 (9.2)	104 (90.8)		0	112 (100)		0	112 (100)	
>18 years old	32 (18)	119 (82)		11 (7.3)	140 (92.7)		13 (8.6)	138 (91.3)	
Swimming pool			0.077 ^a^			0.076 ^a^			0.533 ^a^
Outdoor facility	60 (9.4)	577 (90.6)		16 (2.5)	621 (97.5)		9 (1.4)	628 (98.6)	
Indoor facility	26 (6.3)	384 (93.7)		4 (1)	406 (99)		4 (1)	406 (99)	
Number of training years			<0.001 ^b^			0.005 ^b^			0.001 ^b^
≤3 years	3 (3)	97 (97)		1 (1)	99 (99)		0	100 (100)	
4–6 years	8 (3.1)	254 (96.9)		0	262 (100)		0	262 (100)	
7–9 years	16 (6)	249 (94)		3 (1.1)	262 (98.9)		0	265 (100)	
10–12 years	23 (10.6)	193 (89.4)		9 (4.2)	207 (95.8)		2 (0.9)	214 (99.1)	
>12 years	36 (17.6)	168 (82.4)		7 (3.4)	107 (96.6)		11 (5.4)	193 (94.6)	
Hours of daily training			0.118 ^b^			0.724 ^b^			0.513 ^b^
≤1.5 h/day	18 (5.8)	292 (94.2)		6 (1.9)	304 (98.1)		3(1)	307 (99)	
2 h/day	47 (8.7)	496 (91.3)		9 (1.7)	534 (98.3)		6 (1.1)	537 (98.9)	
>2 h/day	21 (10.8)	173 (89.2)		5 (2.6)	189 (97.4)		4 (2.1)	190 (97.9)	

Values are expressed as n (%), unless stated otherwise. ^a^ X^2^ test, ^b^ X^2^ test for trend.

**Table 5:** Bivariate analyses between Herpes infections and hours of daily training, sunscreen use, and sunburn.

	Herpes Labialis			Herpes Zoster			Genital Herpes		
Characteristic	Yes n (%)	No n (%)	*p* Value	Yes n (%)	No n (%)	*p* Value	Yes n (%)	No n (%)	*p* Value
Hour of daily training			0.007 ^b^			0.760 ^b^			0.183 ^b^
Morning training	3 (16.7)	15 (83.3)		2 (11.1)	16 (88.9)		1 (5.6)	17 (94.4)	
12.00–17.00 p.m.	37 (9.6)	347 (90.4)		7 (1.8)	377 (98.2)		8 (2.1)	376 (97.9)	
After 17.00 p.m.	24 (5.2)	440 (94.8)		5 (1.1)	459 (98.9)		0	464 (100)	
Double training	22 (12.2)	159 (87.8)		6 (3.3)	175 (96.7)		4 (2.2)	177 (97.8)	
Sunscreen use			0.008 ^b^			0.029 ^b^			0.016 ^b^
Always	5 (13.9)	31 (86.1)		2 (5.6)	34 (94.4)		1 (2.8)	35 (97.2)	
Occasionally	20 (13.1)	133 (86.9)		5 (3.3)	148 (96.7)		5 (3.3)	148 (96.7)	
Never	61 (7.1)	797 (92.9)		13 (1.5)	845 (98.5)		7 (0.8)	851 (99.2)	
Sunburn			<0.001 ^a^			<0.001 ^a^			<0.001 ^a^
Yes	18 (20.9)	68 (79.1)		10 (11.6)	76 (88.4)		7 (8.1)	79 (91.2)	
No	68 (7.1)	893 (92.9)		10 (1)	951 (99)		6 (0.6)	955 (99.4)	

Values are expressed as n (%), unless stated otherwise. ^a^ X^2^ test, ^b^ X^2^ test for trend.

## Data Availability

Data are contained within the article.
